# Enhancement of Glucose Uptake by *Meso*-Dihydroguaiaretic Acid through GLUT4 Up-Regulation in 3T3-L1 Adipocytes

**DOI:** 10.3390/molecules22091423

**Published:** 2017-08-28

**Authors:** Anna Lee, Kyeong-Mi Choi, Won-Beom Jung, Heejin Jeong, Ga-Yeong Kim, Ju Hyun Lee, Mi Kyeong Lee, Jin Tae Hong, Yoon-Seok Roh, Sang-Hyun Sung, Hwan-Soo Yoo

**Affiliations:** 1 College of Pharmacy, Chungbuk National University, Osongsaengmyeong 1-ro, Heungduk-gu, Cheongju 28160, Korea; leeanna0719@naver.com (A.L.); mirine0101@hanmail.net (K.-M.C.); jeongwb7@naver.com (W.-B.J.); hheejin110@naver.com (H.J.); kgy5610@naver.com (G.-Y.K.); handrak824@hanmail.net (J.H.L.); mklee@chungbuk.ac.kr (M.K.L.); jinthong@chungbuk.ac.kr (J.T.H.); ysroh@chungbuk.ac.kr (Y.-S.R.); 2 College of Pharmacy, Seoul National University, Seoul 08826, Korea; shsung@snu.ac.kr

**Keywords:** *meso*-dihydroguaiaretic acid, type 2 diabetes mellitus, glucose uptake, GLUT4, adipocyte differentiation

## Abstract

Type 2 diabetes is characterized by insulin resistance, which leads to increased blood glucose levels. Adipocytes are involved in the development of insulin resistance, resulting from the dysfunction of the insulin signaling pathway. In this study, we investigated whether *meso*-dihydroguaiaretic acid (MDGA) may modulate glucose uptake in adipocytes, and examined its mechanism of action. MDGA enhanced adipogenesis through up-regulation of peroxisome proliferator-activated receptor γ and CCAAT/enhancer-binding protein α in 3T3-L1 adipocytes partially differentiated with sub-optimal concentrations of insulin. MDGA also increased glucose uptake by stimulating expression and translocation of glucose transporter 4 (GLUT4) in adipocytes. These results suggest that MDGA may increase GLUT4 expression and its translocation by promoting insulin sensitivity, leading to enhanced glucose uptake.

## 1. Introduction

Type 2 diabetes is characterized by hyperglycemia resulting from insulin resistance [1,2]. The chronic hyperglycemia in diabetes patients leads to dysfunction of different organs such as the heart, blood vessels, eyes, and kidneys [2].

Adipocytes are an important target for the therapy of diabetes mellitus, and adipocyte differentiation has been used for studying anti-diabetic activity [3]. Peroxisome proliferator-activated receptor γ (PPARγ) and CCAAT/enhancer-binding protein α (C/EBPα) play a key role in the complex transcriptional cascade that occurs during adipogenesis, and regulate the metabolic actions of insulin such as glucose transporter 4 (GLUT4) [3,4,5]. Insulin induces the adipocyte differentiation, and leads to the GLUT4 translocation from cytosol to the cell membrane, resulting in glucose uptake [1,2,6].

*meso*-Dihydroguaiaretic acid (MDGA), a major compound found in *Machilus thunbergii* and *Myristica fragrans,* has been reported to have anti-oxidant, anti-inflammatory, and anti-hepatic steatosis activity [7,8,9,10]. MDGA improved the activity of anti-oxidant enzymes such as superoxide dismutase, glutathione peroxidase and catalase, and ameliorated lipid peroxidation by decreasing malondialdehyde production in primary cultures of rat hepatocytes [7]. MDGA inhibited lipid accumulation through AMP-activated protein kinase activation in HepG2 cells, and attenuated hepatic lipid accumulation in high fat diet-induced fatty liver of mice [9,10]. MDGA also inhibited vascular smooth muscle cell proliferation which is a key feature of diverse vascular diseases such as atherosclerosis, hypertension, and cardiovascular disease [11]. However, there are no reports regarding the anti-diabetic activity of MDGA. In this study, we investigated whether MDGA may stimulate glucose uptake by modulating insulin sensitivity in adipocytes.

## 2. Results and Discussion

**Figure 1:**
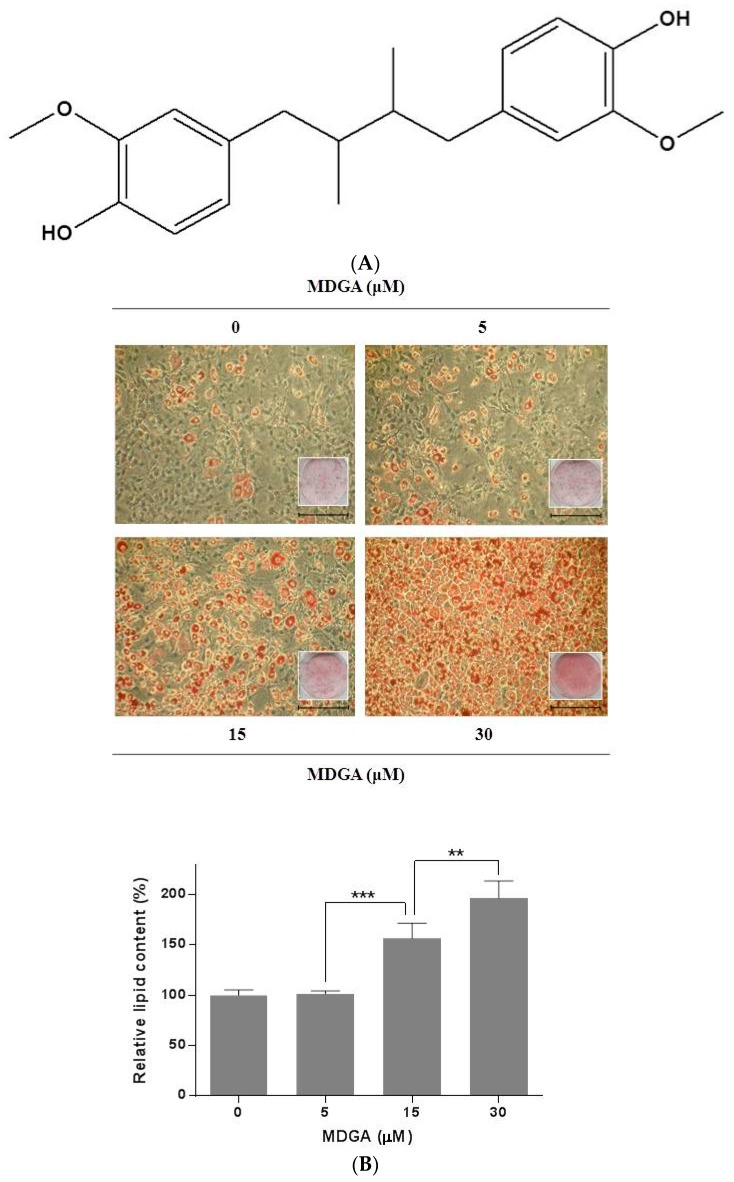
Stimulatory effect of MDGA on adipocyte differentiation. (**A**) Chemical structure of MDGA; (**B**) 3T3-L1 preadipocytes were stimulated with a mixture of adipogenic inducers containing 0.1 μg/mL insulin, 0.5 mM IBMX, and 1 μM dexamethasone. Cells were treated with MDGA (0, 5, 15, or 30 μM) every other day during the first 4 days of differentiation. On day 6, cells were stained with Oil Red O, visualized under a light microscope, and the intensities were quantified. All values are presented as the mean ± SD of three experiments performed in triplicate. Statistical significance: ** *p* < 0.01, *** *p* < 0.001.

Type 2 diabetes mellitus is a metabolic disorder characterized by insulin resistance, resulting in abnormally high glucose levels in the blood [2,12]. Adipogenesis can be initiated by the activation of insulin signaling pathway, and has been used for screening the compounds with anti-diabetic activity [3,13]. *meso*-Dihydroguaiaretic acid (MDGA, Figure 1A) inhibited hepatic lipid accumulation which is a major risk factor for insulin resistance in human HepG2 cells [10,14].

In this study, we examined whether MDGA modulates adipocyte differentiation through activation of the insulin signaling under sub-optimal concentrations of insulin. To verify the effect of MDGA on adipogenesis, 3T3-L1 preadipocytes were differentiated with 0.1 μg/mL insulin plus IBMX and dexamethasone, and simultaneously treated with MDGA (0, 5, 15, or 30 μM). MDGA enhanced the accumulation of lipid droplets, a biomarker of adipocyte differentiation, in a concentration-dependent manner in adipocytes (Figure 1B). The relative lipid content of adipocytes treated with 5, 15, or 30 μM MDGA was enhanced by approximately 1.4%, 56.7%, or 96.6%, respectively, compared with control.

**Figure 2:**
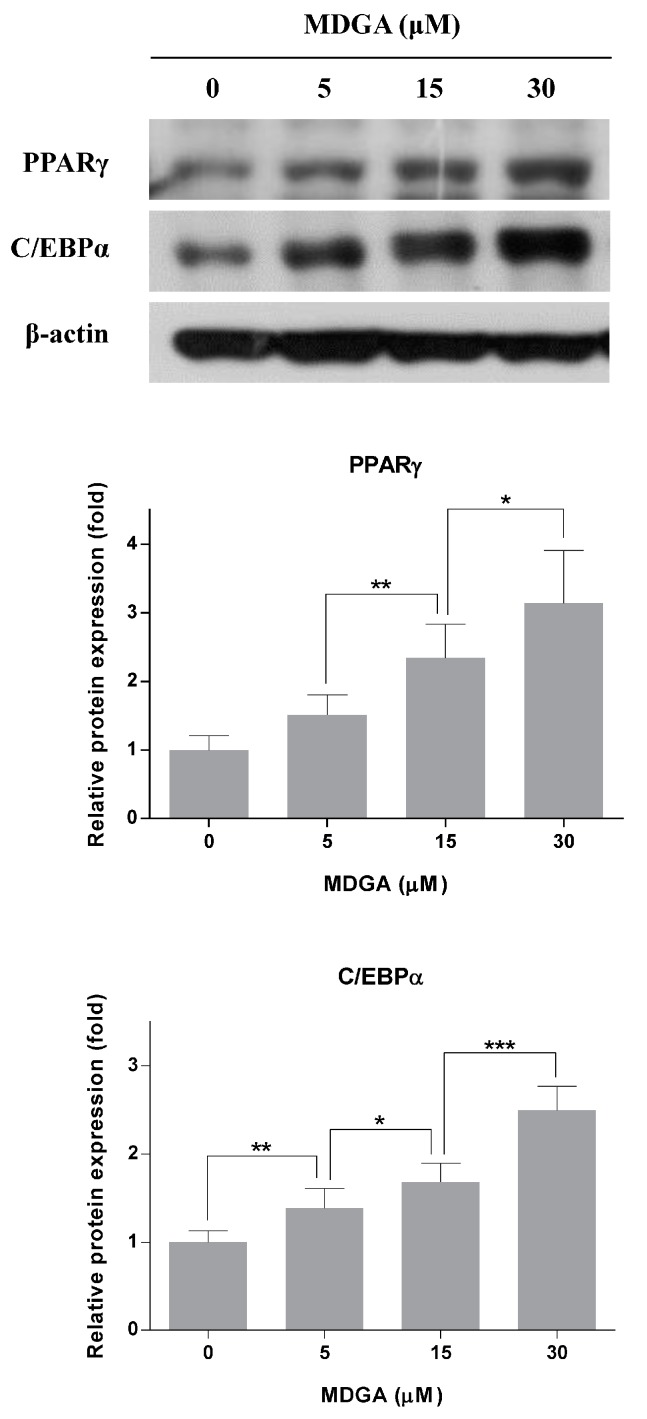
Increased effect of MDGA on PPARγ and C/EBPα expressions. The differentiation of preadipocytes was induced with adipogenic mixture containing 0.1 μg/mL insulin, 0.5 mM IBMX, and 1 μM dexamethasone. Cells were simultaneously treated with MDGA (0, 5, 15, or 30 μM) every other day during the first 4 days of differentiation. On day 6, cells were harvested and the lysates were subjected to Western blot analysis for PPARγ and C/EBPα. The intensity of each band was quantified by the WCIF Image J for Windows Program. All values are presented as the mean ± SD of three experiments performed in triplicate. Statistical significance: * *p* < 0.05, ** *p* < 0.01, *** *p* < 0.001.

PPARγ and C/EBPα are the major transcriptional factors leading to adipocyte differentiation, and regulate glucose metabolism and insulin sensitivity [13,15]. MDGA significantly increased the expression of PPARγ and C/EBPα in adipocytes (Figure 2). PPARγ expression in adipocytes treated with MDGA at 5, 15, or 30 μM was increased by about 1.5-, 2.3-, or 3.1-fold, respectively, compared with control. C/EBPα expression in adipocytes treated with 5, 15, or 30 μM MDGA was increased by ~1.4-, 1.7-, or 2.5-fold, respectively, compared with control. Thus, these results suggest that MDGA enhances adipocyte differentiation through up-regulation of PPARγ and C/EBPα expression by stimulating insulin sensitivity.

**Figure 3:**
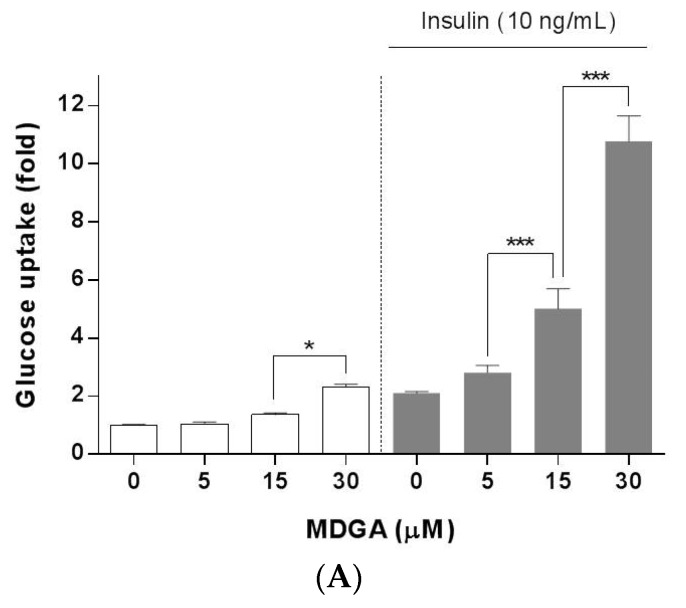

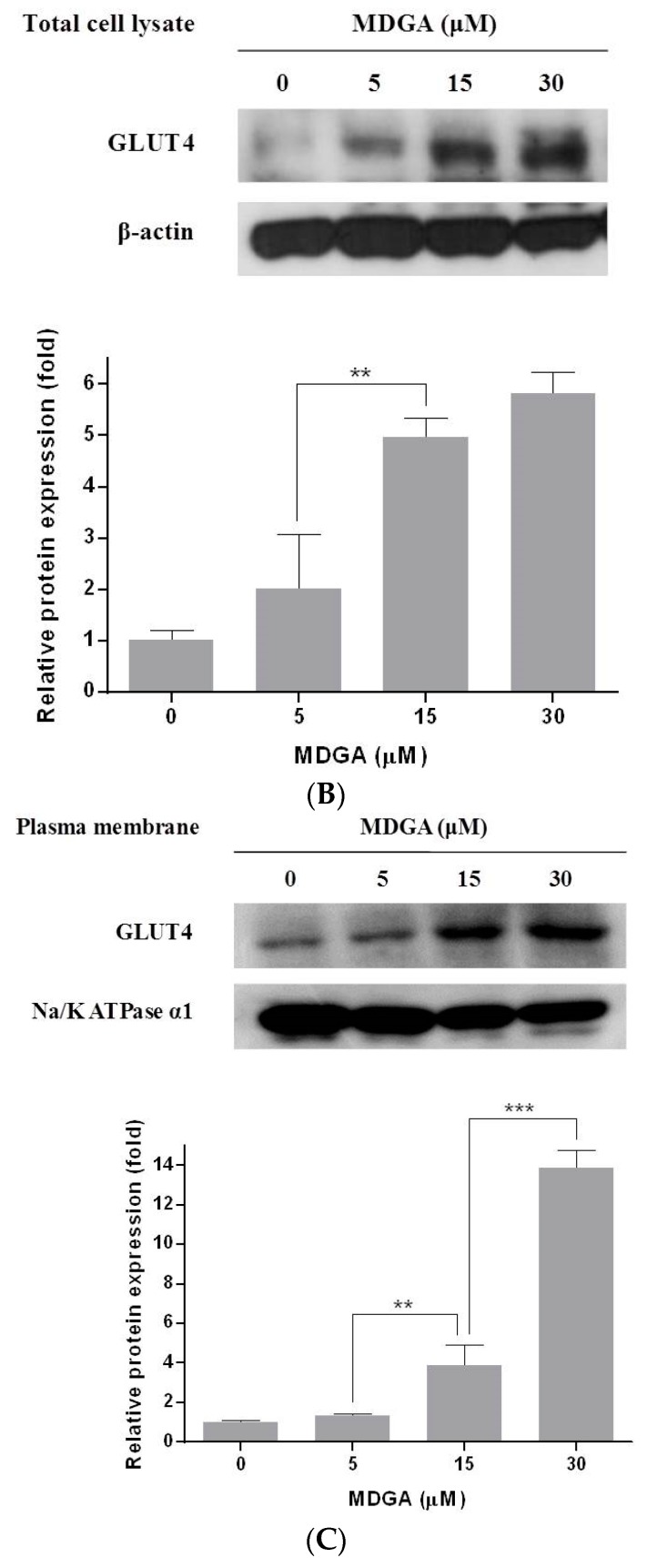
Enhancing effect of MDGA on glucose uptake in adipocytes. 3T3-L1 preadipocytes were stimulated with a mixture of adipogenic inducers containing 0.1 μg/mL insulin, 0.5 mM IBMX, and 1 μM dexamethasone. Cells were simultaneously treated with MDGA (0, 5, 15, or 30 μM) every other day during the first 4 days of differentiation. (**A**) On day 6, the adipocytes were incubated in DMEM containing 0.2% BSA for 4 h, and then glucose uptake was measured. Cells were harvested at day 6, and (**B**) whole cell lysates or (**C**) plasma membrane fractions were subjected to Western blot analysis for GLUT4, and the protein expression levels were normalized against β-actin or Na/K ATPase α1, respectively. The intensity of each band was quantified by the WCIF Image J for Window Program. All values are presented as the mean ± SD of three experiments performed in triplicate. Statistical significance: * *p* < 0.05, ** *p* < 0.01, *** *p* < 0.001.

Glucose uptake in adipose tissue occurs through glucose transporters such as GLUT4 which increases glucose uptake via translocation to the plasma membrane, and is a major mechanism for reducing blood glucose level [16,17,18]. We measured basal and insulin-stimulated glucose uptake in differentiated 3T3-L1 adipocytes. MDGA significantly increased insulin-stimulated glucose uptake in a concentration-dependent manner (Figure 3A). Insulin-stimulated glucose uptake in adipocytes treated with 5, 15, or 30 μM MDGA was significantly increased by approximately 1.3-, 2.4-, or 5.1-fold, respectively, compared with control.

MDGA significantly increased the expression of GLUT4 in whole cell adipocyte lysates (Figure 3B). GLUT4 expression in adipocytes treated with MDGA at 5, 15, or 30 μM was increased by ~2.0-, 5.0-, or 5.8-fold, respectively, compared with control. To determine whether MDGA could induce the GLUT4 translocation, 3T3-L1 cells were treated with MDGA, and the plasma membrane fraction was obtained. MDGA significantly increased the GLUT4 expression in the plasma membrane fraction of adipocytes (Figure 3C). GLUT4 expression in the plasma membrane fraction of adipocytes treated with MDGA at 5, 15, or 30 μM was about 1.3-, 3.9-, or 13.9-fold higher, respectively, than in control. These results suggest that MDGA may enhance glucose uptake in adipocytes via increased GLUT4 expression and translocation.

## 3. Materials and Methods

### 3.1. Reagents

3T3-L1 cells were purchased from the American Type Culture Collection (ATCC, Manassas, VA, USA). Dulbecco’s Modified Eagle’s Medium (DMEM), bovine calf serum (BCS) and fetal bovine serum (FBS) were purchased from Invitrogen (Carlsbad, CA, USA). Insulin and bovine serum albumin (BSA) were obtained from Roche Diagnostics (Mannheim, Germany). 3-Isobutyl-1-methylxanthine (IBMX), dexamethasone, and Oil Red O dye were purchased from Sigma Chemical Co. (St. Louis, MO, USA). 2-Deoxy-D-[1-^3^H(N)] glucose was obtained from American Radiolabeled Chemicals, Inc. (St. Louis, MO, USA). Antibodies against PPARγ, C/EBPα, GLUT4, and β-actin were purchased from Santa Cruz Biotechnology, Inc. (Santa Cruz, CA, USA). Antibodies against Na/K ATPase α1 was obtained from Cell Signaling Technology (Beverly, MA, USA). *meso*-dihydroguaiaretic acid was isolated and characterized from the bark of *Machilus thunbergii* by our research group [19]. All chemicals were of analytical grade.

### 3.2. Cell Culture and Adipocyte Differentiation Induction

3T3-L1 preadipocytes were cultured and differentiated into adipocytes using a method reported previously [20]. Briefly, 3T3-L1 preadipocytes originating from Swiss mouse embryos were cultured in DMEM containing 10% BCS at 37 °C in a 5% CO_2_ incubator. To induce differentiation, 2-day postconfluent preadipocytes were incubated for 2 days in differentiation medium containing 10% FBS, 0.5 mM IBMX, 1 μM dexamethasone, and 0.1 μg/mL insulin. The medium was then changed to DMEM containing 10% FBS and 0.1 μg/mL insulin, and cells were cultured for a further 2 days. Following this, cells were incubated in DMEM supplemented with 10% FBS for 2 more days.

### 3.3. Oil Red O Staining

After the induction of adipocyte differentiation, cells were washed with phosphate-buffered saline (PBS), fixed at room temperature with 10% formalin for 1 h, stained at room temperature with Oil Red O for 1 h, and washed three times with distilled water. For quantitative analysis, Oil Red O stain was dissolved in isopropanol and optical densities were measured using an ELISA reader at 490 nm (Molecular Devices, LLC., Sunnyvale, CA, USA).

### 3.4. Glucose Uptake Assay

Adipocytes were incubated in DMEM containing 0.2% BSA for 4 h and washed two times with Krebs-Ringer Hepes buffer (136 mM NaCl, 4.7 mM KCl, 1.25 mM CaCl_2_, 1.25 mM MgSO_4_, 20 mM Hepes, pH 7.4). The cells were incubated in KRH buffer with or without 10 ng/mL insulin at 37 °C for 15 min. Glucose uptake reaction was initiated by addition of 0.5 μCi/mL 2-deoxy-d-[1-^3^H(N)] glucose as the final concentration in KRH buffer. After 10 min, the cells were quickly washed two times with ice-cold KRH buffer to terminate the reaction. The cells were lysed with 0.5 N NaOH and the radioactivity was determined using a Liquid Scintillation Analyzer (PerkinElmer, Inc., Waltham, MA, USA).

### 3.5. Extraction of Plasma Membrane Protein

Cells were washed with cold PBS and harvested. Plasma membrane protein was extracted using a plasma membrane protein isolation kit (Invent Biotechnologies, Inc., Eden Prairie, MN, USA). The plasma membrane fraction was separated from the cellular components (nuclei, cytosol, and organelles) according to the manufacturer’s instructions. The protein content of plasma membrane was determined with the BCA Protein Assay Reagent (Pierce, Rockford, IL, USA).

### 3.6. Western Blot Analysis

3T3-L1 cells were collected and suspended in a lysis buffer containing 62.5 mM Tris-HCl (pH 6.8), 2% SDS, 10% glycerol, 50 mM dithiothreitol, and protease inhibitor cocktail tablet (Roche Diagnostics, Mannheim, Germany). The total protein concentration of the lysates was determined by using a BCA Protein Assay Reagent (Pierce, Rockford, IL, USA). Proteins in the lysates were electrophoretically separated by 10% SDS polyacrylamide gel and then transferred to a polyvinylidene difluoride membrane (GE Healthcare Life Sciences, Piscataway, NJ, USA). The membranes were blocked in 5% BSA overnight at 4 °C and then incubated overnight at 4 °C with the following primary antibodies: PPARγ (1:1000), C/EBPα (1:1000), GLUT4 (1:1000), Na/K ATPase α1 (1:1000), and β-actin (1:1000). The membranes were incubated with horseradish peroxidase-conjugated secondary antibodies overnight at 4 °C. The bands were visualized with enhanced chemiluminescence (Amersham Pharmacia Biotech, Buckinghamshire, UK) and exposed to X-ray film (Eastman Kodak, Rochester, NY, USA).

### 3.7. Statistical Analysis

All values are expressed as mean ± standard deviation (SD). Statistical significance was determined by one-way analysis of variance with Newman-Keuls Multiple Comparison test. *p*-values less than 0.05 were considered statistically significant.

## 4. Conclusions

**Figure 4:**
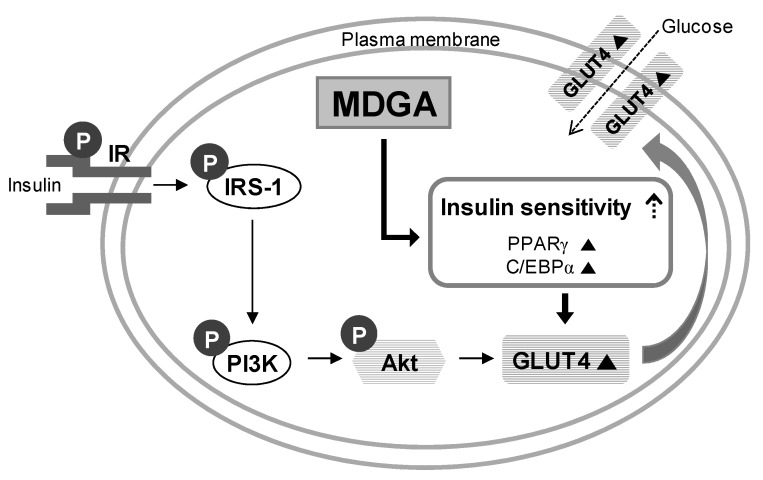
Proposed mechanism of MDGA for enhancement of glucose uptake. MDGA promotes adipocyte differentiation via activation of PPARγ and C/EBPα, and leads to increase glucose uptake into adipocytes through the up-regulation of GLUT4 expression and its translocation. The symbol ▲ represents up-regulation.

MDGA activates PPARγ and C/EBPα, and increases glucose uptake through up-regulation of GLUT4 expression and its translocation by promoting insulin sensitivity in adipocytes (Figure 4).
